# Description of Major Osteoporotic Fractures in Women with Invasive Breast Cancer Who Received Endocrine Therapy

**DOI:** 10.1001/jamanetworkopen.2021.33861

**Published:** 2021-11-17

**Authors:** Joan C. Lo, Cecile A. Laurent, Janise M. Roh, Jean Lee, Malini Chandra, Song Yao, Marilyn L. Kwan

**Affiliations:** 1 Division of Research, Kaiser Permanente Northern California, Oakland, California; 2 The Permanente Medical Group; 3 Department of Cancer Prevention and Control, Roswell Park Comprehensive Cancer Center, Buffalo, New York

## Abstract

This cohort study examines the extent to which pathologic fractures are associated with major osteoporotic fracture events in women with invasive breast cancer who receive endocrine therapy.

## Introduction

Breast cancer remains the most common cancer in US women^1^ and is associated with substantial morbidity, including increased fracture risk attributed in part to estrogen deficiency, aromatase inhibitors, frailty, and skeletal metastases. While fractures associated with these factors have been examined, epidemiologic studies often lack detail regarding pathologic (ie, cancer-related) fractures. We examined the extent to which pathologic fractures are associated with major osteoporotic fracture events in women with invasive breast cancer who received endocrine therapy.

## Methods

This cohort study was approved by the institutional review board at Kaiser Permanente Northern California (KPNC). All study participants provided written informed consent for the Pathways or Research Program on Genes, Environment, and Health (RPGEH) studies. This study followed the Strengthening the Reporting of Observational Studies in Epidemiology (STROBE) reporting guidelines.

The Pathways Study^2^ enrolled women with newly diagnosed invasive breast cancer (ie, diagnosed from 2005 to 2013) at KPNC, of whom 3312 received endocrine therapy.^3^ The RPGEH^4^ identified 1698 women at KPNC with invasive breast cancer that was diagnosed from 1996 to 2014 who received endocrine therapy. This yielded 5010 women who received endocrine therapy followed up from invasive breast cancer diagnosis up to 10 years or until September 30, 2015, for incident fracture.

Breast cancer stage at initial diagnosis was obtained from the KPNC Cancer Registry. *International Classification of Diseases, Ninth Revision* (*ICD-9*) diagnoses of femur fractures (ie, principal hospital diagnoses) and vertebral, humerus, or wrist fractures (ie, hospital, emergency, ambulatory, and institutional-stay diagnoses), including pathologic-coded fractures, were identified from KPNC databases (Table 1). The first fracture per site was adjudicated using clinical, histopathology, and radiology reports to determine incident vs prevalent and pathologic vs nonpathologic fracture. Major osteoporotic fractures were fractures at 1 of these 4 sites. Fracture incidence was calculated with 95% CIs using Poisson distributions. Data were analyzed from 2018 to 2021 using the statistical package SAS version 9.4 (SAS Institute). Statistical significance was set at *P* < .05, and tests were 2-tailed.

**Table 1.** Clinical Adjudication of Major Osteoporotic Fractures in Women With a History of Breast Cancer^a^

Clinical Adjudication	Hip (proximal femur) fracture	Vertebral fracture	Humerus fracture	Wrist fracture
*ICD-9* diagnosis	820.0, 820.2, 820.8 (closed fractures of proximal femur), 733.14, 733.15	805.0, 805.2, 805.4, 805.6, 805.8 (closed vertebral fracture without spinal cord injury), 733.13	812.0, 812.2 (closed fracture of proximal humerus or shaft), 733.11	813.4 (closed fracture of distal radius or ulna), 733.12
Radiology and clinical reports	Fracture/site confirmation: proximal femur (not shaft) Pathologic or nonpathologic	Fracture/site confirmation: cervical, thoracic, lumbar, sacral Pathologic or nonpathologic	Fracture/site confirmation: humerus (not distal) Pathologic or nonpathologic	Fracture/site confirmation: distal radius or ulna Pathologic or nonpathologic

^a^ *International Classification of Diseases, 9th Revision* (*ICD-9*) codes were used to ascertain fracture diagnoses for clinical adjudication. Fracture adjudication was conducted by a physician with expertise in skeletal health supported by a medical records analyst. Incident fractures were identified by acute or subacute characteristics, evidence of ongoing fracture healing, or in the case of vertebral fracture, acute and subacute findings or clinical notes indicating an acute event, presentation, or clearly localizing symptoms. Pathologic (cancer-related) fractures were differentiated from nonpathologic fractures by imaging features, histopathology, evidence of osseous metastases, oncology physician assessment, or targeted treatment (eg, localized radiation therapy). Prevalent vertebral fractures were determined based on previous vertebral fracture history or incidental identification of vertebral fracture of indeterminant age in a patient without an acute event or clearly localizing symptoms. These included a few fractures occurring sometime after breast cancer diagnosis but were prevalent fractures when they were incidentally discovered. Fractures associated with hospitalized major trauma (*ICD-9* E800-E848) were excluded.

## Results

Among 5010 women (mean [SD] age, 60.2 [11.5] years, 543 [10.8%] Asian women, 244 [4.9%] Black women, 473 [9.4%] Hispanic women, 3672 [73.3%] non-Hispanic White women, 78 [1.6%] women of other or unknown ethnicity; 4542 [90.7%] stage I to stage II at initial diagnosis), 340 (6.8%) had incident fracture(s) during follow-up (median [IQR], 6.7 [4.1-9.1] years), including 46 hip, 104 vertebral, 78 humerus, and 137 wrist fractures. These excluded prevalent fractures, which accounted for 46 of 150 (30.7%) identified vertebral fractures. Clinical characteristics by fracture type are shown in Table 2. Among women with hip fracture, 20 (43.5%) were age 80 years or older, compared with less than 25% for women with vertebral (23 [22.1%]), humerus (15 [19.2%]), or wrist fracture (21 [15.3%]) (*P* < .01).

**Table 2.** Characteristics of Women With Invasive Breast Cancer Who Received Endocrine Therapy by Subsequent Incident Fracture Type During Follow-up^a^

Characteristics	No. (%)					
	Hip fracture (N = 46)	Vertebral fracture (N = 104)^**b**^	Humerus fracture (N = 78)	Wrist fracture (N = 137)	Any 1 of 4 fractures (N = 340)	All women (N = 5010)
Age at breast cancer diagnosis, y^c^	^d^ ^,^ ^e^ ^,^ ^f^	^d^				
<65	9 (19.6)	41 (39.4)	40 (51.3)	78 (56.9)	159 (46.8)	3166 (63.2)
65-79	27 (58.7)	50 (48.1)	31 (39.7)	47 (34.3)	143 (42.1)	1623 (32.4)
≥80	10 (21.7)	13 (12.5)	7 (9.0)	12 (8.8)	38 (11.2)	221 (4.4)
Race/ethnicity						
Non-Hispanic White	40 (87.0)	84 (80.8)	67 (85.9)	106 (77.4)	281 (82.7)	3672 (73.3)
Black	<5 (6.5)	5 (4.8)	<5 (2.6)	<5 (2.2)	11 (3.2)	244 (4.9)
Hispanic	<5 (4.4)	6 (5.8)	6 (7.7)	19 (13.9)	30 (8.8)	473 (9.4)
Asian	0	5 (4.8)	<5 (3.8)	8 (5.8)	14 (4.1)	543 (10.8)
Other/unknown	<5 (2.2)	<5 (3.8)	0	<5 (0.7)	<5 (1.2)	78 (1.6)
AJCC stage at breast cancer diagnosis^g^						
Stage I	26 (56.5)	50 (48.1)	43 (55.1)	68 (49.6)	177 (52.1)	2752 (54.9)
Stage II	16 (34.8)	37 (35.6)	26 (33.3)	50 (36.5)	121 (35.6)	1790 (35.7)
Stage III	<5 (6.5)	13 (12.5)	7 (9.0)	17 (12.4)	35 (10.3)	396 (7.9)
Stage IV	<5 (2.2)	<5 (3.8)	<5 (2.6)	<5 (1.5)	7 (2.1)	72 (1.4)
Years from breast cancer diagnosis to fracture, median (IQR)	3.9 (2.3-6.1)	4.2 (2.5-5.9)	3.4 (2.0-5.3)^d^	4.3 (2.7-6.4)	4.0 (2.3-6.0)	NA
Age at fracture, y	^d^ ^,^ ^e^ ^,^ ^f^					
<65	7 (15.2)	30 (28.9)	34 (43.6)	56 (40.9)	121 (35.6)	NA
65-79	19 (41.3)	51 (49.0)	29 (37.2)	60 (43.8)	148 (43.5)	NA
≥80	20 (43.5)	23 (22.1)	15 (19.2)	21 (15.3)	71 (20.9)	NA
Fracture subtype						NA
All women						NA
Nonpathologic	42 (91.3)	82 (78.8)	75 (96.2)	136 (99.3)	312 (91.8)	NA
Pathologic	<5 (8.7)^d^	22 (21.2)^d^	<5 (3.8)^e^	<5 (0.7)	28 (8.2)	NA
Stage I or II at breast cancer diagnosis						
Nonpathologic	39 (92.9)	72 (82.8)	68 (98.6)	118 (100)	281 (94.3)	NA
Pathologic	<5 (7.1)^d^	15 (17.2)^d^	<5 (1.4)^e^	0	17 (5.7)	NA
Stage III or IV at breast cancer diagnosis						
Nonpathologic	<5 (75.0)	10 (58.8)	7 (77.8)	18 (94.7)	31 (73.8)	NA
Pathologic	<5 (25.0)	7 (41.2)^d^	<5 (2.2)	<5 (5.3)	11 (26.2)	NA
Fracture incidence rate per 100 000 person-years during follow-up, (95% CI)						
All fractures	138 (103-184)	314 (259-380)	235 (188-293)	415 (351-490)	1051 (945-1168)	NA
Stage I or II at diagnosis	137 (101-186)	286 (232-353)	226 (179-287)	389 (325-466)	1002 (895-1123)	NA
Stage III or IV at diagnosis	146 (55-390)	629 (391-1012)	330 (171-633)	705 (450-1105)	1596 (1180-2160)	NA
Nonpathologic fractures	126 (93-171)	247 (199-307)	226 (180-283)	412 (348-487)	964 (863-1077)	NA
Stage I or II at diagnosis	128 (93-175)	237 (188-298)	223 (176-283)	389 (325-466)	945 (841-1062)	NA
Stage III or IV at diagnosis	110 (35-340)	370 (199-688)	256 (122-538)	668 (421-1060)	1178 (829-1675)	NA

Abbreviation: NA, not applicable.

^a^ 50.4% received aromatase inhibitors, 21.8% received tamoxifen, 27.8% received both tamoxifen and aromatase inhibitors (eg, one followed by the other).

^b^ For vertebral fractures, the incidence of pathologic vertebral fracture was 66 (44-101) per 100 000 person-years overall, with an incidence of 49 (30-82) per 100 000 person-years for women with stage I or stage II breast cancer at initial diagnosis and 259 (124-543) per 100 000 person-years for women with stage III or stage IV breast cancer at initial diagnosis. Fracture incidence rates are reported per 100 000 person years.

^c^ We estimate that 24% to 28% of women were premenopausal at the time of initial breast cancer diagnosis, based on self-reported premenopausal status in 1001 (30.2%) of 3312 women from the Pathways Study and based on 213 (12.5%) women younger than 50 years or 411 (24.2%) women younger than 55 years of 1698 women from the Research Program on Genes, Environment and Health Study.

^d^ *P* < .05 for any fracture compared with wrist fracture using the χ^2^ test or Fisher exact test.

^e^ *P* < .05 for hip or humerus fracture compared with vertebral fracture using the χ^2^ test or Fisher exact test.

^f^ *P* < .05 for hip fracture compared with humerus fracture using the χ^2^ test or Fisher exact test.

^g^ American Joint Committee on Cancer Tumor, Node, Metastasis Staging System at initial diagnosis.

Pathologic fractures accounted for 22 of 104 (21.2%) of incident vertebral fractures and fewer than 5 of 46 (8.7%) of incident hip fractures (Table 2); the latter is consistent with 10% reported among 752 KPNC women with breast cancer history and hip fracture.^5^ Few humerus and wrist fractures were pathologic. By tumor stage, 15 of 87 (17.2%) vs 7 of 17 (41.2%) vertebral fractures in women with initial stage I and stage II vs stage III to stage IV breast cancer were pathologic (*P* < .05). The incidence of nonpathologic fracture was highest for the wrist, followed by vertebral and humerus fractures, and lowest for hip fractures (Table 2).

## Discussion

This report highlights vertebral fracture considerations among women with breast cancer, where a subset of the sample is prevalent and/or pathologic fractures. Among women with initial stage III or stage IV breast cancer, pathologic fractures comprised at least 1 in 3 subsequent incident vertebral fractures. As the axial skeleton is a common site for breast cancer metastasis and vertebrae a common site for pathologic fracture,^6^ primary care physicians should consider the possibility of pathologic fracture in women with higher risk based on advanced-stage cancer history. Limitations of this study are that our analyses did not account for fracture risk factors, treatment, and chemotherapy and included only clinically diagnosed fractures (asymptomatic vertebral fractures were missed). However, a strength of this study is the large nonreferral population size and comprehensive approach to fracture ascertainment.

Overall, these data support clinicopathologic adjudication of high-risk vertebral fractures and differentiation of prevalent vertebral fractures in studies of women with breast cancer. Examination of nonpathologic fracture risk by treatment type (eg, aromatase inhibitors, cytotoxic chemotherapy) may determine which women benefit from aggressive osteoporotic fracture prevention care.
